# Temporal Changes in Prevalence of Antimicrobial Resistance in 23 U.S. Hospitals

**DOI:** 10.3201/eid0807.010427

**Published:** 2002-07

**Authors:** Scott K. Fridkin, Holly A. Hill, Nataliya V. Volkova, Jonathan R. Edwards, Rachel M. Lawton, Robert P. Gaynes, John E. McGowan, the Intensive Care Antimicrobial Resistance Epidemiology (ICARE), Project Hospitals

**Affiliations:** * Centers for Disease Control and Prevention, Atlanta, Georgia, USA; † Emory University, Atlanta, Georgia, USA

**Keywords:** antibiotic resistance, nosocomial infections, surveillance, epidemiologic methods

## Abstract

Antimicrobial resistance is increasing in nearly all health-care–associated pathogens. We examined changes in resistance prevalence during 1996–1999 in 23 hospitals by using two statistical methods. When the traditional chi-square test of pooled mean resistance prevalence was used, most organisms appear to have increased in prevalence. However, when a more conservative test that accounts for changes within individual hospitals was used, significant increases in prevalence of resistance were consistently observed only for oxacillin-resistant *Staphylococcus aureus*, ciprofloxacin-resistant *Pseudomonas aeruginosa*, and ciprofloxacin- or ofloxacin-resistant *Escherichia coli*. These increases were significant only in isolates from patients outside intensive-care units (ICU). The increases seen are of concern; differences in factors present outside ICUs, such as excessive quinolone use or inadequate infection-control practices, may explain the observed trends.

The increasing prevalence of antimicrobial-resistant organisms, a major public health problem, is of particular concern for hospitals (1,2). However, resistance data aggregated from many hospitals document changes over time but often do not evaluate the consistency of these changes in all the hospitals (3–5). Several statistical tests can be used to evaluate changes in antimicrobial-resistance prevalence; chi-square is commonly used but does not account for consistency of trends in all hospitals. Thus, national or international evaluations based on observed changes in resistance patterns in isolates pooled from all sites can misrepresent the overall trend if a few of the sites report outlier data, as had been observed with data from the National Nosocomial Infections Surveillance system (6). A second difficulty with interpreting data for U.S. trends of antimicrobial resistance in health-care settings is inherent in the diversity of populations served by the facilities.

Monitoring resistance patterns by location within the hospital (e.g., intensive-care units [ICUs], non-ICU inpatient areas, and outpatient areas) can demonstrate substantial changes that would be obscured if hospitalwide data were aggregated into national trends. To determine consistency of changes in antimicrobial-resistance patterns over time in a national monitoring project, we used two statistical methods to evaluate national antimicrobial-resistance data over a 4-year period, as well as assess consistency within hospitals.

## Methods

For this study, we monitored changes in antimicrobial resistance in different hospital areas during two periods (1996–1997 and 1998–1999) in facilities participating in Project ICARE (Intensive Care Antimicrobial Resistance Epidemiology), a joint project of the Hospital Infections Program (now the Division of Healthcare Quality Promotion) of the Centers for Disease Control and Prevention (CDC) and the Rollins School of Public Health of Emory University. Hospitals participating in the ICU surveillance component of the National Nosocomial Infections Surveillance (NNIS) system were invited to participate in the second (January 1996 through December 1997) and third (April 1998 through July 1999) phases of Project ICARE. Twenty-three U.S. hospitals reported acceptable data for both time periods. The surveillance methods and definitions of the NNIS system and Project ICARE have been described (7,8).

Each month, hospitals reported the antimicrobial-susceptibility results of isolates recovered from clinical specimens from patients served by the clinical microbiology laboratory. For study isolates, susceptibility results were reported from all clinical specimens, whether associated with hospital- or community-acquired infection or colonization. Duplicate isolates were excluded; these were defined as isolates of the same organism with the same antimicrobial-resistance pattern recovered from the same patient during a calendar month, regardless of the site of isolation (e.g., blood, sputum, urine, wound). In addition, isolates obtained as part of infection-control surveillance were excluded. When these “surveillance” isolates are excluded, the resistance prevalence (i.e., percent resistant) more closely reflects data routinely aggregated as part of the laboratories’ cumulative susceptibility reports (i.e., cumulative antibiograms). The validity of the susceptibility data has been assessed, and participating laboratories were evaluated as performing reliably. This assessment was done through a proficiency testing program at these laboratories, as well as confirmatory testing of selected isolates (9).

Susceptibility results (MIC and zone diameters) were interpreted according to criteria from the National Committee for Clinical Laboratory Standards (NCCLS) (10–12). The sentinel organisms considered in the analysis, which represented frequently encountered resistance problems in U.S. hospitals, were oxacillin-resistant coagulase-negative staphylococci, oxacillin-resistant Staphylococcus aureus (ORSA), vancomycin-resistant enterococci (VRE), third-generation cephalosporin-resistant Escherichia coli, third-generation cephalosporin-resistant Enterobacter species, ceftazidime-resistant Pseudomonas aeruginosa, ciprofloxacin-resistant P. aeruginosa, ciprofloxacin-resistant E. coli (for E. coli, defined as resistance to either ofloxacin or ciprofloxacin), and third-generation cephalosporin-resistant Klebsiella pneumoniae.

These data were aggregated for each month and stratified by hospital area and time period. To determine temporal trends, we compared all data reported from isolates tested during 1996–1997 (period 1) with all data reported from isolates tested during 1998–1999 (period 2). Data were reported for each hospital area, including each separate ICU (units that provide intensive observation, diagnosis, and therapeutic procedures for critically ill patients); as a pooled total for a given hospital’s non-ICU inpatient areas (areas other than ICUs where the patient stays at least one night in the hospital); and as a pooled total for each hospital’s outpatient areas (urgent care or emergency wards and units that perform same-day surgery or simple diagnostic procedures and therapy, such as chemotherapy, hemodialysis, or cardiac catheterization). Pooled rates were calculated for prevalence of resistance (e.g., percent VRE = proportion of enterococci tested that were resistant to vancomycin) at each hospital. If <10 isolates were tested for antimicrobial susceptibility from a specific hospital area during the study period, the prevalence rate was considered to be of low accuracy, and that hospital area was excluded from further analysis.

To assess the overall magnitude of resistance for each sentinel organism, we calculated an overall (i.e., weighted mean) pooled mean prevalence, combining data from all hospitals, by hospital area and time period. Changes in resistance prevalence over time within each hospital area were assessed by chi-square tests. In addition, each hospital’s change in resistance prevalence over time for each sentinel organism was determined by subtracting the period 2 rate from the period 1 rate. Since the changes in resistance rates for most organisms did not follow a normal distribution, a nonparametric test was used to assess the statistical significance of the temporal changes. The Wilcoxon signed-rank test was chosen for the analysis to take into account the variability of resistance patterns in individual hospitals while minimizing the impact of hospitals with extreme (outlying) values of temporal changes in resistance. The signed-rank test is used to assess the null hypothesis that the population median of the differences in paired observations is equal to zero (13). Since the focus is on medians rather than means, extreme values are less likely to influence the outcome of this test, unlike a t test of the pooled mean values. P values <0.05 were considered significant.

## Results

Of 61 hospitals reporting some data to Project ICARE in either period, 23 (38%) reported at least 6 months of data during both periods and were included in this analysis. Twenty-one (91%) were general hospitals, and 2 (9%) were Veterans Administration hospitals. Fifteen (65%) were affiliated with a medical school. The mean size of participating hospitals was 440 beds (median 356, range 147–1,022); 13 (56%) were in the Atlantic Region, 6 (26%) in the Central Region, 2 (9%) in the New England Region, and 2 (9%) in the Pacific Region. Study hospitals (n=23) did not differ significantly in these characteristics from the ICARE hospitals that were excluded from analysis (n=38) because they submitted data for only one of the two time periods.

The overall pooled mean prevalence of resistance from period 1 to period 2 appeared to have changed for most of the sentinel organisms. The changes were statistically significant when compared by a chi-square test of the pooled means, by time period, for five sentinel organisms in the ICU areas, five in the non-ICU areas, and four in the outpatient areas (Table, footnote). However, when the temporal change in prevalence was evaluated by comparing the median difference in prevalence between period 1 and period 2, no organism demonstrated a significant temporal change in prevalence in the ICUs (Table). In addition, temporal changes remained significant for only three of the sentinel organisms in the non-ICU inpatient area and four in the outpatient area. In non-ICU inpatient areas, significant increases in median resistance rates were noted for ORSA (8.2%), ciprofloxacin-resistant *P. aeruginosa* (3.3%)*,* and ciprofloxacin-resistant *E. coli* (0.6%) (Table). In outpatient areas, significant increases in median resistance rates were evident for ORSA (2.4%), VRE (0.6%), ciprofloxacin-resistant *E. coli* (1.0%), and ciprofloxacin-resistant *P. aeruginosa* (5.0%) (Table). No significant change in resistance prevalence was observed for oxacillin-resistant coagulase-negative staphylococci, third-generation cephalosporin-resistant *E. coli*, third-generation cephalosporin-resistant *Enterobacter* species, or ceftazidime-resistant *P. aeruginosa* in ICU, non-ICU inpatient, or outpatient areas.

**Table:** Weighted pooled mean prevalence and temporal differences of antimicrobial resistance for sentinel organisms, 1996–1999, Project ICARE hospitals

Antimicrobial-Resistant Pathogen	Weighted pooled mean resistance rate (%)		Median difference (%) in resistance rates^a^	N	p-value^b^
	1996–1997	1998–1999			
Intensive-care unit areas					
Oxacillin-resistant CNS	76.0	73.6	-0.01	20	0.8
Oxacillin-resistant *Staphylococcus aureus*	30.9	35.6^c^	1.83	22	0.4
Vancomycin-resistant enterococcus	15.5	15.0	-1.81	20	0.9
Cef3-resistant *Escherichia coli*	0.57	2.2^c^	0.00	20	0.3
Cef3-resistant *Enterobacter* spp.	25.2	25.0	-2.08	17	0.4
Ceftazidime-resistant *P. aeruginosa*	8.3	7.8	0.37	21	0.9
Ciprofloxacin-resistant *P. aeruginosa*	17.7	24.4^c^	0.63	22	0.2
Ciprofloxacin-resistant *E. coli*	0.9	2.0 ^c^	0.00	20	1.0
Cef3-resistant *Klebsiella pneumoniae*	2.4	8.4 ^c^	0.00	18	0.3
Non-intensive–care unit inpatient areas					
Oxacillin-resistant CNS	62.6	63.6	0.41	20	0.6
Oxacillin-resistant *Staphylococcus aureus*	30.2	34.4^c^	8.20	22	0.008
Vancomycin-resistant enterococcus	13.9	11.3	0.93	22	0.4
Cef3-resistant *Escherichia coli*	0.69	0.53	0.00	20	0.9
Cef3-resistant *Enterobacter* spp.	22.1	20.5	-5.90	21	0.4
Ceftazidime-resistant *P. aeruginosa*	5.8	5.9	0.00	21	0.9
Ciprofloxacin-resistant *P. aeruginosa*	17.2	23.9^c^	3.30	22	0.02
Ciprofloxacin-resistant *E. coli*	1.4	2.5^c^	0.57	22	0.008
Cef3-resistant *Klebsiella pneumoniae*	3.6	4.9^c^	0.06	20	0.1
Outpatient/urgent/emergent care patients					
Oxacillin-resistant CNS	45.2	43.6	11.50	21	0.4
Oxacillin-resistant *Staphylococcus aureus*	18.0	22.6^c^	2.40	22	0.009
Vancomycin-resistant enterococcus	2.1	4.8^c^	0.61	21	0.02
Cef3-resistant *Escherichia coli*	0.16	0.23	0.00	22	0.7
Cef3-resistant *Enterobacter* spp.	10.0	9.2	-0.77	21	0.6
Ceftazidime-resistant *P. aeruginosa*	3.8	3.6	0.16	21	0.7
Ciprofloxacin-resistant *P. aeruginosa*	20.0	24.6^c^	5.00	21	0.02
Ciprofloxacin-resistant *E. coli*	0.61	1.4^c^	1.00	22	<0.001
Cef3-resistant *Klebsiella pneumoniae*	1.1	1.5	0.00	20	0.5

^a^Median of the differences in resistance prevalence from period 1 (1996–1997) to period 2 (1998–1999) observed in the (N) hospitals or units reporting resistance information on >10 isolates for each of the time periods. CNS, coagulase-negative Staphylococcus; Cef3, ceftazidime, cefotaxime, or ceftriaxone; for *E. coli,* ciprofloxacin resistance is resistance to either ciprofloxacin or ofloxacin. ^b^p-value by Wilcoxon signed-rank test of the differences at N hospitals or units. ^c^p<0.05 by chi-square test of pooled mean resistance rates between time periods.

## Discussion

These data, which demonstrate a high level of antimicrobial resistance in organisms commonly associated with hospital-acquired infections, are consistent with other reports (3–5,14). However, in this analysis of data from 23 hospitals for 1996–1999, we demonstrate that antimicrobial resistance in the study hospitals has increased consistently for only a few of the sentinel organisms measured. Significant increases were limited to ORSA, ciprofloxacin-resistant *P. aeruginosa*, and ciprofloxacin- or ofloxacin-resistant *E. coli*. Furthermore, these increases were significant only for isolates obtained from non-ICU unit areas. If the traditional chi-square test, which uses the pooled mean prevalence rate, is used to determine the level of significance, significant increases appear to have occurred in most of the organisms studied and throughout the hospital. However, these overall changes in prevalence often were influenced by weighting of the pooled mean by a few hospitals reporting larger numbers of isolates or very large increases in antimicrobial-resistance prevalence. The data from these influential hospitals were not representative of what was observed in most of the hospitals. Thus, the more conservative statistical test used, the Wilcoxon signed-rank test, identified those hospital areas and sentinel organisms where the temporal change was more representative of all the hospitals. By using the more conservative assessment of median differences between time periods, we were able to present a more valid scenario of observations across most of our study hospitals. The paired t test, which tests whether the mean difference in resistance prevalence is equal to zero, is also a viable alternative for analysis of data such as these, provided that sample sizes are large enough to justify the assumption that the differences are normally distributed. If uncertainty exists about the normal distribution, the Wilcoxon signed-rank test is a good choice, since it performs almost as well as the t test when the data are normally distributed.

Although the prevalence of ORSA has not increased in the ICUs of these hospitals, the increase in prevalence of ORSA outside ICUs is very concerning. *S. aureus* is commonly seen with central line-associated bloodstream or surgical site infection (15). The median increase of 2.4% in isolates from the outpatient areas is approximately a 10% increase over the baseline prevalence observed in the first time period (i.e., 20% ORSA). Although these isolates are mostly from emergency room patients who likely have had recent exposures to health-care settings, this prevalence rate is comparable with the rate of 20%–23% observed in hospitalized patients in the early 1990s (16). With more frequent reports of community-onset ORSA infections (17–19), we expect this prevalence rate to continue to increase unless adequate prevention measures are identified and implemented.

Gram-negative bacilli are frequently associated with hospital-acquired infections, particularly ventilator-associated pneumonia and catheter-associated urinary tract infections (15,20). Although antimicrobial resistance in these organisms to third-generation cephalosporins is of great clinical concern (4,21,22), no consistent increases occurred in prevalence of third-generation cephalosporin resistance in *E. coli*, *Enterobacter* spp., *K. pneumoniae*, or *P. aeruginosa*. This finding does not imply that some hospitals did not experience significant increases, but rather that changes over time were not consistent between facilities in all hospital areas. This observation may reflect successful infection-control strategies in study hospitals, but further study is needed to validate this conjecture. For *K. pneumoniae* or *E. coli*, these data suggest that ESBL-producing *K. pneumoniae* or *E. coli* remains a focal problem.

The data are strikingly different for ciprofloxacin resistance in *P. aeruginosa* and *E. coli*. With these organisms, resistance from non-ICU patient isolates and outpatient isolates increased across all hospitals, but resistance in the ICU patient isolates did not increase significantly. Contributing factors may include the large amounts of quinolones used by patients outside the ICU or the development of ciprofloxacin resistance in *P. aeruginosa* unrelated to the ICU setting (23).

No consistent increases in resistance were observed in ICU isolates for any of the study organisms, which may reflect successful infection-control programs in the ICUs at these study hospitals. However, this finding also might reflect a variation in the evolution of antimicrobial resistance at these hospitals. For example, the prevalence of antimicrobial resistance increased first in the ICUs, and factors similar to those in the ICUs have moved into the non-ICU areas, resulting in increases in these areas during the second half of the study.

One major limitation of this study is the small size of this national sample. With only 23 hospitals reporting sufficient data in each study period, inferences from these data about the direction of antimicrobial resistance in the United States overall must be made with caution. Although these hospitals are representative of all NNIS hospitals, hospitals in the Mid- and South-Atlantic regions are overrepresented (24). However, statistically significant trends of increasing resistance for ORSA and ciprofloxacin-resistant *P. aeruginosa* or *E. coli,* found using a conservative test for significance, suggest that these changes are consistent in all study hospitals. This finding may indicate that these resistant organisms represent problems faced by most U.S. hospitals.

Another limitation is lack of confirmation of the clinical relevance of the organisms evaluated in this study, which represent organisms associated both with colonization and infection. However, we minimized inclusion of colonizing organisms by eliminating duplicate reports. In addition, in a separate analysis of these surveillance data, we have demonstrated that the cumulative susceptibility reports generated from these data are comparable with those for organisms reported to be associated with definitive hospital-acquired infection (25). Therefore, we believe the data in this study are representative of the susceptibilities of the organisms associated with hospital-acquired infections.

These data suggest that monitoring antimicrobial resistance by hospital area can identify national trends in resistance prevalence affecting only certain hospital areas. Increases are also widespread in study hospitals in patients outside the ICU. Attention should be paid to identifying novel measures for curbing increases in antimicrobial resistance outside ICUs and to assessing why current measures are failing.

Aggregated susceptibility data, such as those presented here, may be easily obtained as part of local or regional surveillance efforts. Written guidelines for producing cumulative susceptibility reports from hospital-based surveillance efforts have been created by the National Committee for Clinical Laboratory Standards (26). Public health authorities can use such data produced by standard specifications to assess trends in prevalence of antimicrobial-resistant organisms associated with health-care delivery. However, analysis of temporal trends should include assessing consistency of changes in the facilities under surveillance by using appropriate statistical tests.

## References

[1] McGowan JE Jr, Tenover FC. Control of antimicrobial resistance in the health care system. Infect Dis Clin North Am. 1997;11:297–311. 10.1016/S0891-5520(05)70357-3 9187948

[2] Schwartz B, Bell D, Hughes JM. Preventing the emergence of antimicrobial resistance: a call for action by clinicians, public health officials, and patients. JAMA. 1997;278:944–5. 10.1001/jama.278.11.944 9302249

[3] Deikema DJ, Pfaller MA, Schmitz FJ, Smayevsky J, Bell J, Jones RN, Survey of infections due to Staphylococcus species: frequency of occurrence and antimicrobial susceptibility of isolates collected in the United States, Canada, Latin America, Europe, and the Western Pacific Region for the SENTRY Antimicrobial Surveillance Program, 1997–1999. Clin Infect Dis. 2001;32:S114–32. 10.1086/320184 11320452

[4] Gales AC, Jones RN, Turnidge J, Rennie R, Ramphal R. Characterization of *Pseudomonas aeruginosa* isolates: occurrence rates, antimicrobial susceptibility patterns, and molecular typing in the Global SENTRY Antimicrobial Surveillance Program, 1997–1999. Clin Infect Dis. 2001;32:S146–55. 10.1086/320186 11320454

[5] Low DE, Keller N, Barth A, Jones RN. Clinical prevalence, antimicrobial susceptibility, and geographic resistance patterns of enterococci: results from the SENTRY Antimicrobial Surveillance Program, 1997–1999. Clin Infect Dis. 2001;32:S133–45. 10.1086/320185 11320453

[6] Monnet D, Biddle JW, Edwards JR, Culver DH, Tolson JS, Martone WJ, Evidence of interhospital transmission of extended-spectrum β-lactam-resistant *Klebsiella pneumoniae* in the United States, 1986–1993. Infect Control Hosp Epidemiol. 1997;18:492–8. 9247832 10.1086/647654

[7] Emori G, Culver DH, Horan TC, Jarvis WR, Olson DR, Banerjee S, National nosocomial infections surveillance (NNIS): Description of surveillance methods. Am J Infect Control. 1991;19:19–35. 10.1016/0196-6553(91)90157-8 1850582

[8] Fridkin SK, Steward CD, Edwards JR, McGowan JE Jr, Culver DH, Gaynes RP, Surveillance of antimicrobial use and antimicrobial resistance in U.S. hospitals: Project ICARE Phase 2. Clin Infect Dis. 1999;29:245–52. 10.1086/520193 10476720

[9] Steward CD, Wallace D, Hubert SK, Lawton R, Fridkin SK, Gaynes RP, Ability of laboratories to detect emerging antimicrobial resistance in nosocomial pathogens: a survey of Project ICARE laboratories. Diagn Microbiol Infect Dis. 2000;38:59–67. 10.1016/S0732-8893(00)00161-9 11025185

[10] National Committee for Clinical Laboratory Standards. Performance standards for antimicrobial disk susceptibility tests. NCCLS approved standard M2–A6. Wayne (PA): The Committee; 1997.

[11] National Committee for Clinical Laboratory Standards. Methods for dilution antimicrobial susceptibility tests for bacteria that grow aerobically. NCCLS approved standard M7-A4. Wayne (PA): The Committee; 1997.

[12] National Committee for Clinical Laboratory Standards. Performance standards for antimicrobial susceptibility testing. NCCLS approved standard M100-S6. Wayne (PA): The Committee; 1995.

[13] Mendenhall W, Wackerly DD, Schaeffer RL. Mathematical statistics with applications. Belmont (CA): PWS-KENT Publishing Company; 1990.

[14] Pfaller MA, Jones RN, Messer SA, Edmond MB, Wenzel RP. National surveillance of nosocomial blood stream infection due to *Candida albicans*: frequency of occurrence and antifungal susceptibility in the SCOPE Program. Diagn Microbiol Infect Dis. 1998;31:327–32. 10.1016/S0732-8893(97)00240-X 9597393

[15] Fridkin SK, Welbel SF, Weinstein RA. Magnitude and prevention of nosocomial infections in the intensive care unit. Infect Dis Clin North Am. 1997;11:479–96. 10.1016/S0891-5520(05)70366-4 9187957

[16] Fridkin SK, Gaynes RP. Antimicrobial resistance in intensive care units. Clin Chest Med. 1999;20:303–16. 10.1016/S0272-5231(05)70143-X 10386258

[17] Boyce JM. Are the epidemiology and microbiology of methicillin-resistant *Staphylococcus aureus* changing? JAMA. 1998;279:623–4. 10.1001/jama.279.8.623 9486761

[18] Centers for Disease Control and Prevention. Four pediatric deaths from community-acquired methicillin-resistant *Staphylococcus aureus*—Minnesota and North Dakota, 1997–1999. MMWR Morb Mortal Wkly Rep. 1999;48:707–10. 21033181

[19] Herold BC, Immergluck LC, Maranan MC, Lauderdale DS, Gaskin RE, Boyle-Vavra S, Community-acquired methicillin-resistant *Staphylococcus aureus* in children with no identified predisposing risk. JAMA. 1998;279:593–8. 10.1001/jama.279.8.593 9486753

[20] Centers for Disease Control and Prevention. National Nosocomial Infections Surveillance (NNIS) report, data summary from January 1990–May 1999. Am J Infect Control. 1999;27:520–32. 10.1016/S0196-6553(99)70031-3 10586157

[21] Karas JA, Pillay DG, Muckart D, Sturm AW. Treatment failure due to extended spectrum β-lactamase. J Antimicrob Chemother. 1996;37:203–4. 10.1093/jac/37.1.203 8647770

[22] Rice LB, Eckstein EC, DeVente J, Shlaes DM. Ceftazidime-resistant *Klebsiella pneumoniae* isolates recovered at the Cleveland Department of Veterans Affairs Medical Center. Clin Infect Dis. 1996;23:118–24. 8816140 10.1093/clinids/23.1.118

[23] McCaig LF, Hughes JM. Trends in antimicrobial drug prescribing in office-based physicians in the United States. JAMA. 1995;273:214–9. 10.1001/jama.273.3.214 7807660

[24] Richards C, Emori G, Edwards JR, Fridkin SK, Tolson JS, Gaynes RP, Characteristics of hospitals and infection control professionals participating in the National Nosocomial Infections Surveillance System 1999. Am J Infect Control. 2001;29:400–3. 10.1067/mic.2001.118408 11743488

[25] Fridkin SK, Edwards JR, Tenover FC, Gaynes RP, McGowan JE Jr. Antimicrobial resistance prevalence rates in hospital antibiograms reflect prevalence rates in pathogens associated with hospital-acquired infections. Clin Infect Dis. 2001;33:324–30. 10.1086/321893 11438897

[26] National Committee for Clinical Laboratory Standards. M39 analysis and presentation of cumulative antimicrobial susceptibility test data. The Committee. 2002. In press.

